# Case Study on Shifts in Human Skin Microbiome During Antarctica Expeditions

**DOI:** 10.3390/microorganisms13112491

**Published:** 2025-10-30

**Authors:** Kyu-Chan Lee, Hanbyul Lee, Ok-Sun Kim, Woo Jun Sul, Hyeonah Lee, Hye-Jin Kim

**Affiliations:** 1 Department of Systems Biotechnology, College of Biotechnology & Natural Resource, Chung-Ang University, Anseong 17546, Republic of Korea; 2 Division of Life Sciences, Korea Polar Research Institute, Incheon 21990, Republic of Korea; 3 Department of Dermatology, Chung-Ang University College of Medicine, Seoul 06974, Republic of Korea; 4 Division of Zoonotic and Vector Bone Disease Research, Center for Infectious Disease Research, Korea National Institute of Health, Cheongju 28159, Republic of Korea

**Keywords:** skin microbiome, Antarctic expeditions, environmental and lifestyle factors, microbial diversity, resilience and adaptability

## Abstract

The human skin microbiome plays a crucial role in maintaining skin health by acting as a barrier against pathogens and modulating immune regulation. This case study investigates the skin microbiome of two healthy Korean male individuals in their 20s during Antarctic expeditions, focusing on microbial changes, reversion to pre-expedition states, and the influence of environmental and lifestyle factors. Notable microbial alterations were observed, including increases in Pseudomonadota and decreases in Actinomycetota, indicating pronounced microbial shifts in response to harsh environmental factors such as low temperature and humidity. Post-expedition revealed incomplete recovery to pre-expedition states, with Host A showing a higher resilience index, suggesting faster microbial recovery. Correlation analyses revealed associations between microbial changes and environmental factors (e.g., temperature, humidity, atmospheric pressure) as well as lifestyle factors (e.g., sunblock usage, outdoor activities), highlighting complex interactions between host behaviors and microbiome dynamics. Despite the study’s limited sample size, these findings offer insights into the adaptability and resilience of the skin microbiome under extreme environments, with potential implications for health management and skincare strategies during isolated and prolonged expeditions.

## 1. Introduction

The human skin microbiome, a vital ecosystem of bacteria, fungi, and viruses, is fundamental to skin health by acting as a barrier against pathogens, aiding wound healing, and regulating immune responses [1,2,3]. The composition and diversity of the skin microbiome are influenced by external factors (e.g., temperature, humidity, and sunlight [4]) and internal factors (e.g., age, gender, living environment, and hygiene) [5,6,7]. Antarctica, with its extreme cold, low humidity, high ultraviolet (UV) radiation, and strong winds, presents a unique natural laboratory to study microbial adaptations under environmental stress [8,9]. Recent evidence indicates that temperatures in Antarctica are increasing, accelerating the dynamics of native species, including microbial communities highly sensitive to temperature changes [9,10]. These environmental stressors—reduced humidity, extreme cold, and increased UV radiation (which can cause DNA damage in host and microbial cells [11])—pose significant challenges to the health and resilience of human skin microbiomes. Korea’s two Antarctic research stations, King Sejong Station on King George Island (62°13′ S, 58°47′ W) and Jang Bogo Station in Terra Nova Bay (74°37′ S, 164°13′ E), are located in distinct climatic regions, providing unique environments for investigation [12,13,14,15,16]. These contrasting environments offer an opportunity to investigate how such extreme conditions influence human-associated microbial communities, including the skin microbiome.

Studies of the human microbiome in extreme environments have revealed consistent patterns of microbial adaptation and dysbiosis. In spaceflight contexts, ground-based simulations such as the MARS500 study documented changes in gut microbiome composition during 520 days of confinement, including alterations in the Bacteroidota-to-Bacillota ratio, shifts in dominant taxa, and altered functional capacity in energy metabolism, with reversibility observed upon return to Earth [17]. The NASA Twins Study similarly demonstrated significant alterations in microbial community structure and taxonomic abundance during actual spaceflight [18]. High-altitude expeditions have shown altitude-dependent microbiome shifts, with *Prevotella copri* emerging as the most enriched taxon in populations ascending to high altitude, while *Faecalibacterium* and *Blautia* decreased with rising altitude [19]. These high-elevation environments also exhibited distinct diversity patterns: alpha diversity metrics (Shannon diversity and observed OTUs) decreased with increasing elevation across body sites, while beta diversity showed an increasing trend with elevation [20]. Antarctic expedition research revealed that the journey’s stressors induced significant alterations within the gut microbiome, including a sharp decrease in diversity, highlighting the gut’s sensitivity to the combined physiological and psychological pressures of such expeditions [21]. UV radiation exposure, a prominent feature of polar environments, has been shown to induce specific compositional changes in the skin microbiome, with increases in Cyanobacteria, Fusobacteriota, and Verrucomicrobiota phyla, while Lactobacillaceae and Pseudomonadaceae families decreased [22]. These findings collectively highlight the vulnerability of human microbiomes to alterations under extreme environmental conditions, characterized by reduced microbial diversity, shifts in dominant taxa, and potential links to host immune and metabolic functions [17,18,19,20,21]. However, despite extensive research on gut microbiomes in extreme conditions, there remains a notable gap in understanding the skin microbiome specifically during Antarctic expeditions.

This study addresses this gap by investigating the skin microbiome of two healthy individuals during Antarctic expeditions, focusing on three key questions: (i) How do skin microbial compositions change during the expedition? (ii) To what extent do they return to pre-expedition states? (iii) What environmental and lifestyle factors drive these changes? Understanding these changes is critical for developing practical applications, such as skin microbiome-based health management strategies for polar explorers or astronauts. Additionally, these findings could inform the design of skincare products tailored for extreme environments, offering insights into the resilience and adaptability of the human skin microbiome.

## 2. Materials and Methods

### 2.1. Expedition Routes and Skin Swap Sample Collection

**Figure 1:**
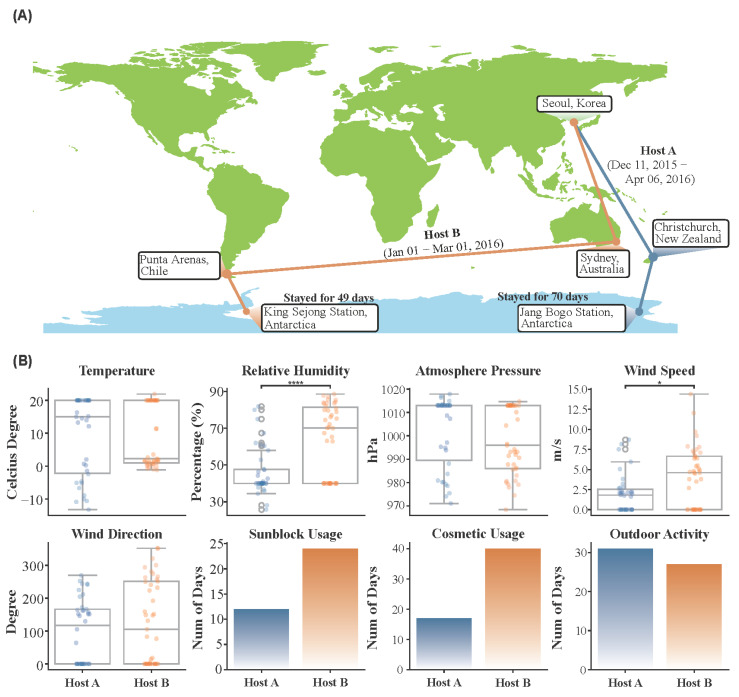
Geographic routes and external factors of two Korean individuals during the Antarctic expeditions. (**A**) Travel routes (blue: Host A; orange: Host B) and stay durations of Host A and Host B during their Antarctic expeditions. Host A traveled from Seoul (South Korea) to Jang Bogo Station (Antarctica) and stayed for 70 days, while Host B traveled to King Sejong Station and stayed for 49 days. (**B**) Comparison of environmental (temperature, relative humidity, atmospheric pressure, wind speed, and wind direction) and lifestyle factors (sunblock usage, cosmetic usage, and outdoor activity) experienced by both hosts. * *p* < 0.05, **** *p* < 0.0001.

In 2015 and 2016, two healthy Korean male hosts individually participated in Antarctic expeditions (Figure 1A). Host A (20s, male, 4-month expedition) traveled by air from Seoul (Republic of Korea) to Christchurch (New Zealand) and then proceeded to Jang Bogo Antarctic Research Station (Terra Nova Bay, Antarctica) aboard the research vessel ‘Italica’. Host A stayed in Christchurch for three days and on the vessel for two weeks. After about two months of stay at Jang Bogo Station, Host A returned to Seoul by sailing from Jang Bogo Station to Christchurch aboard the research vessel ‘Araon’, followed by a flight to Seoul.

Host B (20s, male, 2-month expedition) flew to King Sejong Station (Barton Peninsula, King George Island, Antarctica) via Sydney (Australia) and Punta Arenas (Chile). Host B stayed in Sydney for a day and in Punta Arenas for two days. After approximately one month at King Sejong Station, Host B returned to Seoul along the same route.

During the entire Antarctic expeditions, the hosts collected time-series cheek swab samples every 2–3 days using sterilized swabs (CLASSIQSwabs^TM^ Standard Fiber Wrapped Swabs 168C, Copan Diagnostics Inc., Murrieta, CA, USA). The swab samples were stored at −81 °C until DNA extraction. On each sampling day, the hosts recorded a lifestyle log detailing their use of sunblock and cosmetics and whether they had engaged in outdoor activities. The hosts recorded their logs whenever they collected their samples.

The study was conducted in accordance with the Declaration of Helsinki, and the protocol was approved by Chung-Ang University College of Medicine Institutional Review Board, Seoul, Republic of Korea (P1504-034, 24 April 2015). Written informed consent was obtained from all participants prior to sample collection. All samples were anonymized, and no identifiable personal information was included in the manuscript.

### 2.2. DNA Extraction and Amplicon Sequencing

The heads of each swab sample were transferred to new screw-capped tubes, and genomic DNA was extracted using the PureLink^®^ Genomic DNA Mini Kit (Invitrogen, Carlsbad, CA, USA). Following the Gram-positive bacterial cell lysate protocol, lysozyme and a lysozyme digestion buffer (25 mM Tris-HCl, pH 8.0, 2.5 mM EDTA, 1% Triton X-100) was added to the samples and briefly vortexed to facilitate bacterial cell lysis. After incubating at 37 °C for one hour, 45 μL of proteinase K and 445 μL of genomic lysis/binding buffer were added. Using flame-sterilized forceps, two 5 mm steel beads (Qiagen, Redwood City, CA, USA) were placed in each sample. Bead beating was then performed for one minute using the Bead Beater 16 (Bio Spec Products Inc., Bartlesville, OK, USA). The samples were cooled on ice for 10 min and subsequently incubated at 55 °C for 30 min. Genomic DNA was extracted with 30 μL of PureLink^®^ Genomic Elution Buffer after two DNA washing steps. The purity and concentration of the extracted DNA were evaluated using the NanoDrop 2000 (Thermo Fisher Scientific Inc., Waltham, MA, USA). The genomic DNA was then sent to Macrogen Inc. (Seoul, Republic of Korea) for amplicon analysis (targeting the V4-V5 hypervariable regions of the 16S rRNA genes) using the Illumina MiSeq platform.

### 2.3. Metadata Preprocessing

The weather data history used in this study was downloaded from the Korean Polar Data Center (KPDC), of which the entry IDs are KOPRI-KPDC-00000538, KOPRI-KPDC-00000543, KOPRI-KPDC-00000625, and KOPRI-KPDC-00000643. These datasets included several environmental parameters, such as temperature (°C), relative humidity (%), atmospheric pressure (hPa), wind speed (m/s), and wind direction (deg), where 0° represents north and values increase clockwise. The missing metadata were filled from the Meteostat (https://meteostat.net/en/; accessed on 12 April 2024).

Environmental factors were handled based on the “Outdoor activity” status. If “Outdoor activity” was false, the factors were filled with predefined constants according to NIST.IR.8250 (National Institute of Standards and Technology) [23]: temperature was set to 20 °C, relative humidity was set to 40%, atmospheric pressure to 1013 hPa, and both wind speed and wind direction were set to 0. Subsequently, the missing values were filled using interpolation. To normalize the data for subsequent analysis, standard scaling was applied using the “StandardScaler” class from scikit-learn Python library (version 0.24.1) [24]. This scaling method transformed the data to have a mean of 0 and a standard deviation of 1. This ensured that all environmental parameters were on a consistent scale, allowing for more reliable comparisons and reducing the influence of different units or ranges on the analysis.

### 2.4. Skin Microbiome Analysis

The skin microbiome analysis was performed using the QIIME2 (version 2023.9) platform [25]. The raw sequence data underwent quality control via the DADA2 pipeline [26], which included denoising to correct sequencing errors and removing chimeric sequences to ensure data accuracy. An amplicon sequence variant (ASV) feature table was generated, representing the unique sequences present in the samples. Features in the ASV table with an occurrence greater than 0.1 and a total count exceeding 5 were selected for further analysis. Taxonomic classifications were assigned to the ASVs using the Greengenes2 database (version 2022.10) [27], matching the ASVs to known bacterial taxa for detailed microbial composition analysis at various taxonomic levels, from phylum to species. A multiple sequence alignment of the ASVs was created using MAFFT (Multiple Alignment using Fast Fourier Transform; version 7.520) [28]. Subsequently, a rooted phylogenetic tree was constructed using FastTree and the “root_at_midpoint()” method from the “TreeNode” class in the scikit-bio package (version 0.5.9) [29]. This tree represents the evolutionary relationships among the ASVs, which are crucial for subsequent diversity analyses.

Both alpha and beta diversity metrics were calculated using the scikit-bio package to evaluate microbial diversity within samples and between samples, respectively. Alpha diversity was assessed using metrics such as Chao1 (an estimator of species richness) [30], observed features (a measure of the total number of distinct ASVs observed in a sample) [31], the Shannon index (a measure of diversity accounting for both richness and evenness) [30], and the Simpson index (a measure of diversity giving more weight to common or dominant species) [30]. Beta diversity was analyzed using the Bray–Curtis distance matrix, which measures the differences between microbial communities based on both abundance and composition of ASVs. Principal Coordinates Analysis (PCoA) was then performed to visualize the differences in microbial communities between samples.

### 2.5. Removal of Batch Effects

Batch effects were corrected using a custom script implemented in Python (version 3.8.15). This step minimized variations due to batch processing, allowing for more accurate comparisons between samples. For the input data, a pseudo-count was added to zero values to avoid undefined behavior during logarithmic transformation. Specifically, the minimum non-zero value in the dataset was identified, and half of this value was added to the zero entries. The adjusted data matrix was then log-transformed (log10). Batch correction was performed using the pyCombat [32] function with covariate adjustment. Metadata exhibiting multicollinearity were removed beforehand to prevent confounding effects. The corrected data were exponentiated to return to the original scale. Then, batch correction was validated by Uniform Manifold Approximation and Projection (UMAP) [33], allowing for the assessment of corrected data distribution and the effectiveness of batch effect removal. Relative abundance was subsequently calculated by normalizing the feature counts in each sample.

### 2.6. Statistical Analysis

Alpha diversity metrics (Chao1, observed features, Shannon index, and Simpson index) were utilized to evaluate the diversity within individual samples. The Kruskal–Wallis test, a non-parametric method, was employed to determine statistical differences among groups. This test was chosen due to its robustness in handling non-normally distributed data typical of microbiome studies. Post hoc comparisons were conducted using the Bonferroni correction to adjust for multiple comparisons, ensuring that the likelihood of type I errors (false positives) was minimized.

Beta diversity, which assesses differences in microbial community composition between samples, was analyzed using PERMANOVA (Permutational Multivariate Analysis of Variance) [34]. This method, implemented in the scikit-bio package, evaluates whether the observed differences between groups are statistically significant, accounting for the complex multivariate nature of microbiome data. The PERMANOVA results were notated within PCoA plots, which provided a clear representation of the differences in microbial communities across the samples.

The resilience index, which quantifies the ability of the skin microbiome to recover its baseline community composition after environmental disturbances. The index was computed for each stage of the trip by quantifying how skin microbial community composition changed relative to the initial baseline (BeforeTrip). For each trip status, the dissimilarity from the baseline community was calculated at two points: at the start (*D*_L_) and the end (*D*_N_) of each status. These dissimilarity values were derived from the Bray–Curtis distance matrix, representing the degree to which microbial community composition differed from its pre-expedition state. The period of time for each stage was calculated by determining the difference in days between the first (*T*_L_) and last (*T*_N_) sampling points of each trip status. The resilience index for each trip status was computed using the following formula [35]:

$$Resilience=\;\frac{2\;\left|\;D_{0}-D_{L}\;\right|}{\left|\;D_{0}-D_{L}\;\right|+\left|\;D_{0}-D_{N}\;\right|-1}\;\times \;\frac{1}{\left(\;T_{N}-\;T_{L}\right)\;in\;days}$$

This formula captures the degree to which the skin microbial community diverged from and then returned to its baseline state, normalized by the time taken for these changes to occur. A higher resilience index indicates a quicker or more complete return to the baseline state, while a lower index suggests prolonged or incomplete recovery from environmental disturbances.

Using MaAsLin2 (version 1.16.0) [36] with default settings, we analyzed the microbial abundance across different trip statuses to identify which microbial taxa showed significant variations in abundance. This approach allowed us to assess how specific environmental and lifestyle changes during the trip influenced the composition of the skin microbiome. The MaAsLin2 analysis highlighted key taxa whose abundance fluctuated significantly between different trip phases, providing insight into the dynamic responses of microbial communities to external stressors and environmental conditions encountered during the expedition.

To explore the relationships between beta diversity and various environmental (e.g., temperature, atmospheric pressure, and relative humidity) and lifestyle factors (e.g., sunblock usage, cosmetic usage, and outdoor activity), both continuous and categorical data were used. Correlation analyses were performed using Spearman’s rank correlation coefficient for continuous data and the point-biserial correlation coefficient for binary categorical data, using the “scipy” package (version 1.13.1) [37]. Subsequently, the correlations between the diversity and the factors were visualized by Python visualization library, such as matplotlib (version 3.9.2) [38] and seaborn (version 0.13.2) [39].

## 3. Results

### 3.1. Environmental and Lifestyle Characteristics of Hosts During Antarctic Expedition

The environmental and lifestyle differences between Host A and Host B during the Antarctic expedition revealed significant differences that may have influenced their skin microbiomes (Figure 1B). External environmental factors such as temperature, relative humidity, atmospheric pressure, and wind conditions were compared between the two hosts. Among these, relative humidity and wind speed were significantly different between Host A and Host B (*p* < 0.05). Host B experienced significantly higher relative humidity (median: 70.2%) and wind speed (median: 4.6 m/s) compared to Host A (median relative humidity: 40%; median wind speed: 2.1 m/s). Median temperature differences (Host A: 13.71 °C, Host B: 2.2 °C) were not statistically significant. These results reflect the distinct environmental exposures faced by each host.

Host A operated in a relatively stable environment, whereas Host B was exposed to more dynamic and variable conditions. Although both hosts engaged in a similar number of days with outdoor activity, the nature of these activities differed significantly. Host B’s regimen involved more prolonged and physically demanding outdoor tasks, suggesting greater overall exposure to environmental stressors that may affect skin microbial composition. Consistent with this higher exposure, lifestyle-related factors also varied. Host B reported more frequent use of sunblock and cosmetics than Host A. These differences in the intensity of outdoor exposure and corresponding personal skincare routines, particularly in UV protection and chemical exposure, likely contributed to variations in their skin microbiomes by altering surface conditions.

### 3.2. Skin Microbial Communities in Expedition Hosts During Trips to Antarctica

**Figure 2:**
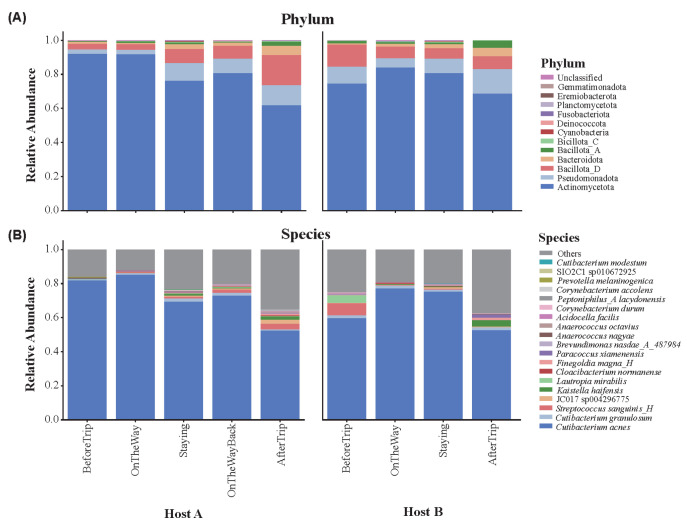
Temporal shifts in bacterial composition at the phylum and species levels for Host A and Host B during the Antarctic expeditions. (**A**) Relative abundance of bacterial taxa at the phylum level for Host A and Host B across different stages of the trip (BeforeTrip, OnTheWay, Staying, OnTheWayBack, AfterTrip). (**B**) Relative abundance of bacterial taxa at the species level for both hosts across the same stages.

The skin microbial compositions of Host A and Host B were compared across multiple taxonomic levels during their Antarctic expeditions (Figure 2; Table 1). At the phylum level, the dominant groups in both hosts were Actinomycetota, Bacillota_D, and Pseudomonadota, with Actinomycetota being the most prevalent. On average, Actinomycetota accounted for 78.14% of the microbial community in Host A and 80.2% in Host B throughout the entire expedition. Pseudomonadota and Bacillota_D represented smaller proportions, each contributing around 6–8% in both hosts. While Actinomycetota decreased over time in Host A, dropping from 91.97% before the trip to 61.88% after the trip, Pseudomonadota increased from 2.65% to 11.90%. In Host B, Actinomycetota showed an increase during the trip but decreased slightly after returning. Pseudomonadota followed a consistent increasing trend, rising from 5.44% (OnTheWay) to 14.41% after the trip.

At the class level (Figure S1A; Table S1), Actinomycetia (affiliated to phylum: Actinomycetota), Bacilli (phylum: Bacillota), and Gammaproteobacteria (phylum: Pseudomonadota) were the most dominant classes. Host A experienced a decrease in Actinomycetia and an increase in Bacilli and Gammaproteobacteria after the trip. Host B followed a similar pattern, with Actinomycetia increasing during the trip but decreasing afterward, and Gammaproteobacteria rising consistently.

At the genus level (Figure S1B; Table S2), the dominant genera included *Cutibacterium* (phylum: Actinomycetota), *Corynebacterium* (phylum: Actinomycetota), *Streptococcus* (phylum: Bacillota), and *Staphylococcus* (phylum: Bacillota). Both hosts experienced a decline in *Cutibacterium* after the trip, while *Streptococcus* increased sharply in Host A but decreased in Host B. *Corynebacterium* showed an initial decrease during the trip in both hosts, followed by an increase afterward.

At the species level (Figure 2B; Table S3), *Cutibacterium acnes* (*C. acnes*) was the most prevalent species, following similar trends as the genus level. *Streptococcus sanguinis_H* (*S. sanguinis_H*) increased in Host A after the trip, while it decreased significantly in Host B. All detailed relative abundance for each level was provided in Supplementary Tables.

### 3.3. Skin Microbiome Diversity by Trip Status

**Figure 3:**
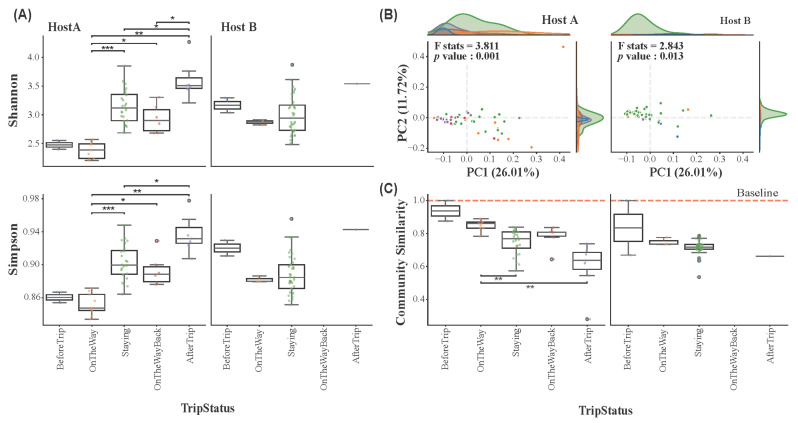
Alpha and beta diversity of microbial communities in Host A and Host B during the Antarctic expedition. (**A**) Alpha diversity (Shannon and Simpson indices) of microbial communities for Host A and Host B, grouped by TripStatus (BeforeTrip: blue; OnTheWay: orange; Staying: green; OnTheWayBack: red; AfterTrip: purple). Significant differences are indicated with *p*-values (* *p* < 0.05, ** *p* < 0.01, *** *p* < 0.001). (**B**) Bray–Curtis-based Principal Coordinate Analysis (PCoA) illustrating the variation in microbial community composition for both hosts across different trip statuses. Significant differences between groups are denoted by PERMANOVA (F statistics and *p*-values). (**C**) Boxplots for microbial community shifts across the trip statuses, relative to baseline (BeforeTrip).

The impact of travel on the alpha diversity of skin microbial communities in Host A and Host B was assessed across five different trip statuses: BeforeTrip (Seoul, Republic of Korea), OnTheWay, Staying (Antarctica), OnTheWayBack, and AfterTrip (Seoul, Republic of Korea). Alpha diversity indices, including Shannon, Simpson, Chao1, and Observed Features (Figure 3A and Figure S2), revealed distinct patterns between the two hosts. Beta diversity was also analyzed to assess differences in microbial community composition between trip statuses (Figure 3B). A statistical test of beta diversity was conducted using PERMANOVA (Permutational Multivariate Analysis of Variance) to evaluate the significance of changes in microbial community structure across the different trip stages.

Statistical analysis using the Kruskal–Wallis test with post hoc Bonferroni correction revealed significant changes in alpha diversity across trip statuses for both hosts. As shown in Figure 3A, Host A exhibited a noticeable increase in Shannon and Simpson indices during the stay in Antarctica, indicating the influence of the extreme environment on the skin microbiome. Although alpha diversity declined during the return phase (OnTheWayBack), this change was not statistically significant. A significant increase was observed post-trip. On the other hand, Host B exhibited a decrease in alpha diversity during their stay in Antarctica, with the alpha indices increasing after the trip. However, these changes were not statistically significant, indicating that the skin microbiome remained relatively stable throughout the trip, despite slight fluctuations in Host B. For Chao1 and Observed Features, Host A displayed similar patterns to the Shannon and Simpson indices. Host B, however, showed an increase in species richness during the Antarctic stay, though without statistical significance (Figure S2).

Distinct shifts in the microbial communities were observed in Host A (Figure 3B), which demonstrates clustering of microbial communities according to the different trip statuses (F-statistic = 3.811, p < 0.05). Visually, the PCoA plot shows the most pronounced separation for samples from the Antarctic stay, which form a distinct cluster away from the pre-expedition (BeforeTrip) samples. Despite some recovery after returning from the expedition, the microbial communities after the trip did not fully revert to the pre-trip (BeforeTrip) baseline, indicating lasting changes in the skin microbiome in Host A. These results suggest that the skin microbiome in Host A underwent substantial alterations during the Antarctic expedition. In contrast, Host B exhibited less distinct clustering of microbial communities across the trip statuses compared to Host A, but the microbial communities still showed significant changes throughout the expedition (F-statistic = 2.843, p < 0.05).

### 3.4. Host-Specific Skin Microbiome Resilience in Response to Environmental Stress

To assess skin microbiome resilience, we first analyzed how microbial community similarity to the pre-expedition baseline changed over time (Figure 3C). The community similarity to the baseline decreased for both hosts during the expedition, reaching its lowest point during their stay in Antarctica. This indicates a significant divergence from their original microbial state, confirming that the Antarctic environment acted as a major disturbance. To quantify the rate of these changes and the capacity for recovery, we then calculated a resilience index (RI) for each trip phase (Figure S3). For Host A, the initial recovery rate was slow (OnTheWay, RI = 0.17) and was severely impeded during the Antarctic stay, with the RI dropping to 0.04. This low rate is consistent with the low and declining community similarity observed in Figure 3C during the same period. The recovery rate improved during the return journey (OnTheWayBack, RI = 0.16) and rebounded sharply after returning to the home environment (AfterTrip, RI = 1.03), indicating a strong post-expedition recovery capacity. In contrast, Host B initially exhibited a very rapid recovery rate (OnTheWay, RI = 1.44). However, this resilience was not sustained, as the RI plummeted to 0.04 during the Antarctic stay. This collapse in recovery speed directly mirrors the dramatic drop in community similarity seen in Figure 3C. Post-expedition recovery could not be assessed due to a lack of data.

Taken together, these findings highlight contrasting recovery trajectories. Host A demonstrated a recovery pattern that was severely impeded by the Antarctic environment before rebounding upon return, whereas Host B exhibited an initial rapid recovery followed by a pronounced decline in resilience under sustained stress. The low community similarity and RI values for both hosts during the Staying stage underscore the significant disturbances, limiting the ability of their skin microbiomes to recover or maintain pre-expedition community structures. These results emphasize the differential resilience capacities of microbial communities to extreme environmental stressors and provide insights into the adaptability of host-associated microbiomes during environmental perturbations.

### 3.5. Correlation of Environmental and Lifestyle Factors and Skin Microbiome Changes

**Figure 4:**
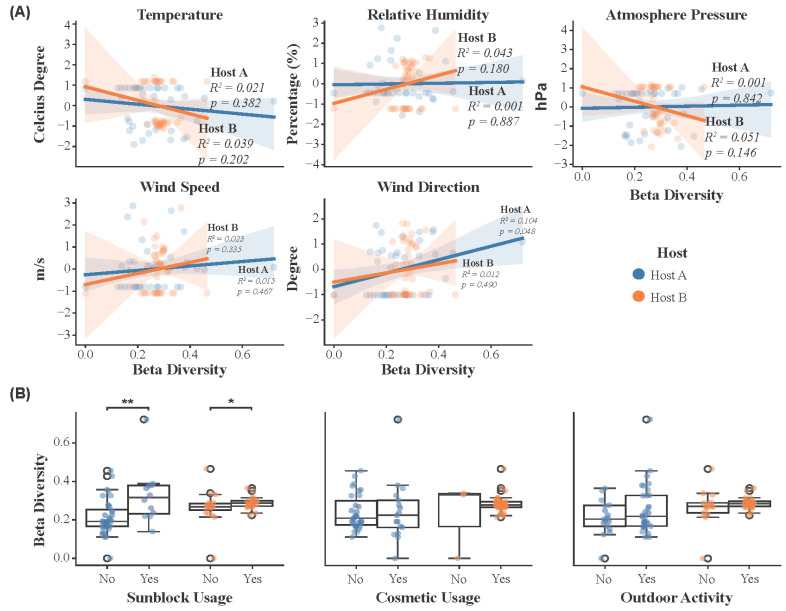
Correlation between the microbial community changes and environmental/lifestyle factors for Host A and Host B. (**A**) Linear regression showing the relationship between beta diversity and environmental factors, including temperature, relative humidity, atmospheric pressure, wind speed, and wind direction. (**B**) Boxplots for the correlation between beta diversity and the impact of lifestyle factors (e.g., sunblock usage, cosmetic usage, and outdoor activity). Significant differences are indicated; * *p* < 0.05, ** *p* < 0.01.

To investigate the impact of environmental and lifestyle factors on the magnitude of microbial community shift over time, we performed linear regression analysis on continuous environmental variables (Figure 4A) and compared beta diversity across categorical lifestyle factors (Figure 4B). Environmental variables included temperature, relative humidity, atmospheric pressure, wind speed, and wind direction, while lifestyle factors included sunblock usage, cosmetic usage, and outdoor activity.

For Host A, a significant positive correlation was identified between beta diversity and wind direction (R^2^ = 0.104, p < 0.05; Figure 4A), indicating that changes in wind direction had a measurable impact on the skin microbiome during the expedition. In contrast, temperature, relative humidity, atmospheric pressure, and wind speed showed no significant correlations, suggesting they had little effect on skin microbial communities in Host A. For Host B, no significant correlations were observed between beta diversity and any of the environmental factors, implying that their skin microbial communities in Host B were less affected by environmental changes compared to Host A.

We also evaluated the relationship between beta diversity and lifestyle factors, including sunblock usage, cosmetic usage, and outdoor activity, based on binary (Yes/No) responses. In both hosts, sunblock usage was significantly associated with differences in beta diversity (p < 0.05; Figure 4B), indicating a measurable impact on the skin microbial composition. In contrast, cosmetic usage and outdoor activity did not show significant effects in either host. This suggests that these factors, such as environmental exposure during temporal outdoor activities, might not significantly alter the microbial community structure on the skin.

### 3.6. Temporal Shifts and Environmental Correlations of Key Skin Microbes

**Figure 5:**
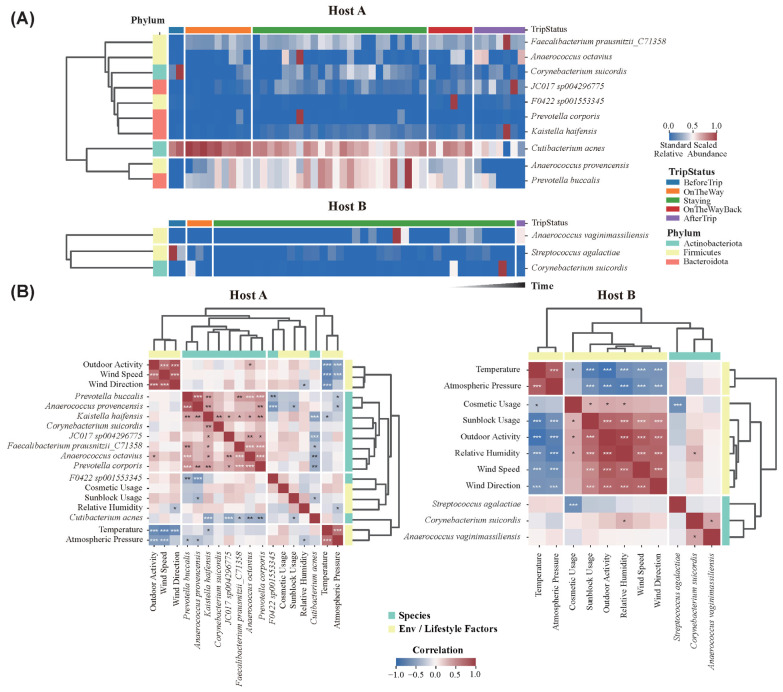
Temporal changes in significant microbial species and their correlations with environmental and lifestyle factors in Host A and Host B. (**A**) Heatmaps showing the temporal changes in relative abundance of significant microbial species identified via MaAsLin2 analysis. Time points and TripStatus are represented along the horizontal axis, with color intensity indicating the relative abundance of each species over time. (**B**) Correlation matrix depicting the relationship between significant microbial species and environmental/lifestyle factors. Positive correlations (red) and negative correlations (blue) are shown with *p*-values; * *p* < 0.05, ** *p* < 0.01, *** *p* < 0.001.

MaAsLin2 analysis revealed significant temporal changes in the relative abundance of 12 key microbial species during the Antarctic expedition (Figure 5A). In Host A, species such as *C. acnes* showed a gradual decrease throughout the expedition, while *A. provencensis* and *P. buccalis* increased in abundance during the Staying phase in Antarctica, reflecting their response to the extreme environmental conditions. Post-expedition, their abundances returned closer to pre-trip levels. Other microbial species, such as *F. prausnitzii* and *A. Octavius*, showed moderate increases in abundance compared to the pre-trip levels. In Host B, species such as *A. vaginimassiliensis*, and *C. suidordis* exhibited increased abundances during and after the expedition compared to the BeforeTrip phase.

The correlation between these microbial species and environmental/lifestyle factors was assessed using Spearman’s rank and Point-Biserial correlation packages (Figure 5B). While strong correlations were observed among various environmental factors, associations between microbial species and environmental/lifestyle factors were generally weak in both hosts. In host A, several significant correlations between specific species and environmental factors were identified. Notably, *P. buccalis* (R = −0.293, p < 0.05) and *A. provencensis* (R = −0.327, p < 0.05) showed significant negative correlations with atmospheric pressure, suggesting reduced prevalence under higher pressure conditions. Similarly, *K. haifensis* demonstrated a significant negative correlation with temperature (R = −0.3, p < 0.05), indicating increased prevalence at lower temperatures. Significant correlations were also observed with lifestyle factors; for instance, *C. acnes* (R = −0.304, p < 0.05) and *A. provencensis* (R = −0.363, p < 0.05) were negatively associated with sunblock usage, which implies that these species were less prevalent on the skin of individuals who regularly used sunblock. In Host B, *S. agalactiae* showed a significant negative correlation with cosmetic usage (R = −0.677, p < 0.05), while *C. suicordis* showed a positive correlation with relative humidity (R = 0.309, p < 0.05). These results underscore the differential influence of environmental and lifestyle factors on skin microbial composition across individuals.

### 3.7. Representative Skin Microbes Are Influenced by Environmental and Lifestyle Factors During Antarctic Expedition

In this study, we identified six microbial species predominantly found on human skin [40,41,42] from our dataset: *Cutibacterium acnes* (*C. acnes*), *Cutibacterium modestum* (*C. modestum*), *Cutibacterium granulosum* (*C. granulosum*), *Corynebacterium amycolatum_A* (*C. amycolatum*), *Streptococcus sanguinis_H* (*S. sanguinis*), and *Micrococcus luteus* (*M. luteus*). These species were selected for further analysis due to their established presence as part of the normal skin microbiota.

The 5-point moving average of relative abundance revealed distinct temporal trends across the expedition stages (Figure S4A). Species such as *C. acnes*, *C. modestum*, *C. granulosum*, *C. amycolatum_A*, *S. sanguinis_H*, and *M. luteus* exhibited fluctuations in abundance over time, with the 5-point moving average highlighting broader trends. Notably, *C. acnes* in Host A showed a gradual decline throughout the expedition, while in Host B, it demonstrated more dynamic fluctuations. Similarly, *S. sanguinis* in Host A displayed an increasing trend, whereas in Host B, its abundance decreased over time. The correlation was also analyzed between these microbial species and environmental/lifestyle factors (Figure S4B). In Host A, *C. acnes* exhibited a significant negative correlation with sunblock usage (R = −0.30, p < 0.05), indicating that application of sunblock may suppress its growth in this host. Conversely, no significant correlation was observed between cosmetic usage and *C. acnes* abundance (R = −0.10, p > 0.05), suggesting that cosmetics did not substantially impact the presence of this species in Host A. In Host B, however, *C. acnes* demonstrated a significant positive correlation with cosmetic usage (R = 0.48, p < 0.05), indicating that cosmetic use may have promoted the growth of this species. No significant positive correlation was detected between sunblock usage and *C. acnes* abundance (R = 0.17, p > 0.05) in Host B, implying that sunblock did not significantly influence the species in this host. *C. amycolatum_A* and *S. sanguinis_H* in Host A showed positive correlations with various environmental and lifestyle factors, such as wind speed and humidity; however, these correlations were not statistically significant (p > 0.05). In contrast, these skin microbes exhibited significant negative correlations with cosmetic usage (R_C.amycolatum_A_ = −0.34, R_S.sanguinis_H_ = −0.49, p < 0.05) in Host B, suggesting that cosmetic use may have contributed to a decrease in the abundance of these species in this host.

## 4. Discussion

This study provides a detailed case analysis of how the skin microbiome responds to extreme environmental stressors during Antarctic expeditions, emphasizing inter-individual differences in microbial dynamics, resilience, and influencing factors. Antarctica presents one of the most extreme natural environments on Earth, with low temperatures, strong winds, low humidity, and high UV exposure [43,44,45,46]. These factors are known to exert significant pressure on human physiology and microbial ecosystems. Our findings reveal marked differences in the microbial responses of two hosts (Host A and Host B) to Antarctic conditions, underscoring the importance of host-specific and environmental factors in shaping the skin microbiome. Both individuals exhibited an increase in Pseudomonadota during the expedition, consistent with this phylum’s known adaptability to harsh environmental conditions, including low humidity and high UV radiation [47,48,49,50]. However, Host A experienced a notable decrease in Actinomycetota, alongside increases in Pseudomonadota and Bacillota, suggesting greater microbial disruption. In contrast, Host B’s microbiome showed smaller compositional shifts, indicative of higher microbiome stability [47]. These contrasting responses likely reflect differences in local environmental conditions—such as temperature and humidity between Jang Bogo and King Sejong stations—as well as variations in personal habits, such as sunblock and cosmetic usage.

Interestingly, despite experiencing greater microbial disruption during the expedition, Host A demonstrated a high resilience index after the expedition, returning to a microbial composition similar to the pre-trip status. This suggests a robust recovery capacity, potentially mediated by physiological factors such as skin barrier integrity, immune status, or genetic predisposition [35,51,52,53]. In contrast, a resilience index could not be calculated for Host B due to insufficient post-expedition samples, representing a limitation in cross-host comparisons. Understanding microbial resilience in extreme conditions is essential for developing strategies to protect and maintain microbiome stability during prolonged stays in isolated environments, such as polar expeditions or space travel [54]. Future research could focus on identifying the molecular and ecological mechanisms driving microbial resilience, as well as testing interventions like probiotics or protective skin treatments to enhance microbiome stability under extreme stress. The impact of site-specific environmental factors also emerged as a critical variable. Host A, located at Jang Bogo Station, faced drier conditions (median RH: 40%) compared to Host B at King Sejong Station (median RH: 70.2%). Although Host B experienced a colder median outdoor temperature, the pronounced dryness faced by Host A appears to have been the more significant environmental stressor, contributing to the more dynamic microbiome shifts observed in this host. Prior research has shown that extreme environments, including high altitude [20] and space missions [55,56,57], can cause significant shifts in human microbiomes. Similarly to those findings, our results suggest that Antarctic-specific stressors, such as low relative humidity and increased UV radiation, may drive microbial restructuring, favoring taxa better adapted to environmental stress, such as Pseudomonadota [4,58,59].

While this study focused on the cheek, an area directly exposed to the environment, it is plausible that these extreme Antarctic stressors would elicit distinct responses across other skin regions with varying ecological characteristics. For instance, sebaceous sites like the forehead and nose, which are typically dominated by the lipophilic *C. acnes*, might have experienced an even more pronounced decline in this taxon. The extreme cold and low humidity likely reduce sebum production, creating a less favorable environment for these commensals. In contrast, the hands, which experience the most direct contact with environmental surfaces and aerosols, could be hypothesized to show a greater influx of transient environmental microbes, such as soil-derived Pseudomonadota, leading to a more variable and less stable community structure compared to the face. Conversely, occluded and moist areas like the axilla, which are largely buffered from external environmental shifts, would likely exhibit far greater microbial stability. Investigating these diverse skin niches in future expeditions would provide a more holistic understanding of how the human skin microbiome adapts to extreme environments on a whole-body scale.

The observed shifts in microbial composition, particularly the decrease in Actinomycetota and the increase in Pseudomonadota, may have significant consequences for skin immune homeostasis and barrier function. A stable commensal skin microbiome is crucial for maintaining barrier integrity by promoting the expression of tight junction proteins and regulating epidermal differentiation [41]. The dysbiosis observed could therefore imply a compromised skin barrier, potentially leading to increased trans-epidermal water loss (TEWL)—a critical concern in the low-humidity Antarctic environment. Furthermore, key commensals such as *S. epidermidis* are known to modulate innate immunity by inducing the production of antimicrobial peptides (AMPs) and fine-tuning inflammatory responses [60]. A disruption of this delicate balance, as suggested by our data, could diminish the skin’s intrinsic capacity to resist colonization by opportunistic pathogens, thereby increasing susceptibility to infections and potentially impairing wound healing processes during prolonged expeditions where medical support is limited.

Evidence from animal models further supports these potential physiological implications. Studies in mice, for instance, have demonstrated that the skin microbiota broadly modulates host gene expression, directly influencing pathways related to innate immunity and epidermal barrier function [61]. A plausible mechanistic hypothesis for our findings involves the concept of sterile inflammation. In an environment like Antarctica, severe physical stressors such as extreme cold and UV radiation can cause direct cellular damage, leading to the release of Damage-Associated Molecular Patterns (DAMPs) from host skin cells, even in the absence of infection [62]. These DAMPs can trigger innate immune receptors, inducing a state of low-grade, sterile inflammation. This inflammatory microenvironment would inevitably alter host-microbe crosstalk, shifting the ecological niche to favor stress-tolerant opportunistic taxa, like many within Pseudomonadota, over the established resident commensals. This DAMP-driven mechanism provides a plausible link between the harsh Antarctic environment and the specific microbial shifts documented in our study, greatly enhancing the biological relevance of our findings.

In addition to the direct environmental stressors, Antarctic aerosols, soil microbes, and physical activity may have influenced the skin microbiome. Although Antarctica is often regarded as having a pristine atmosphere, microbial aerosols have been detected and may have transiently interacted with the skin microbiome [63,64,65]. For instance, the increase in certain taxa, such as Pseudomonadota, could be partially attributed to the deposition of aerosolized microbes onto the skin [66]. Nevertheless, the ability of these exogenous microbes to integrate into the resident microbial community is likely constrained by host defense mechanisms, such as low skin pH and antimicrobial peptides [40,59], suggesting that their impact may be temporary or minimal. Similarly, the unique microbial communities in Antarctic soils may have also played a role [67,68]. While these findings highlight the potential contribution of environmental microbes to the observed changes, their impact appears to be limited.

Similarly, the unique microbial communities in Antarctic soils, populated by extremophilic microbes adapted to cold temperatures and nutrient-poor conditions, may have also played a role in shaping the skin microbiome [67,68]. Physical contact with soil or wind-driven dispersion of soil microbes may transiently deposit these organisms onto the skin. However, their ability to significantly alter the established microbial community is constrained by the competitive nature of resident microbes and the robust defense mechanisms of the host skin. While these findings highlight the potential contribution of environmental microbes to the observed changes, their impact appears to be limited. Further studies are needed to quantify the extent of aerosol and soil microbial deposition on the skin and to elucidate the conditions under which these external microbes might integrate into the skin microbiome.

The differences in environmental conditions and lifestyle habits between Host A at Jang Bogo Station and Host B at King Sejong Station also appear to have significantly influenced the microbial shifts observed in their skin microbiome. Host A was subjected to more extreme environmental conditions, like lower temperature and lower relative humidity, compared to the conditions experienced by Host B at King Sejong Station. These harsher conditions likely contributed to the more pronounced microbial shifts observed in Host A, particularly the decrease in Actinomycetota and the increase in Pseudomonadota, which are known to respond differently to environmental stressors. As observed in a previous study [58], a reduction in Actinomycetota and an increase in Pseudomonadota have been reported under extreme conditions such as flooding and waterlogging. This pattern may also manifest in polar environments, where Pseudomonadota are better adapted to extreme stress conditions. Host B, despite exposure to high wind speeds, may have benefited from more frequent use of sunblock and cosmetics, which can form physical and chemical barriers that mitigate environmental stressors [4,69]. These products may influence skin surface characteristics like hydration and pH, thereby altering microbial colonization patterns. Although both hosts had a similar number of outdoor activity days, Host B engaged in activities of longer duration and higher intensity, which may have led to greater cumulative UV exposure and perspiration, influencing microbial shifts differently than in Host A. While these factors were not directly measured, they may have contributed to microbial shifts by influencing the skin’s microenvironment. Overall, the interplay between environmental factors and individual behaviors appears to have played a key role in shaping the distinct microbial responses observed between the two hosts. Future studies should aim to quantify these unmeasured factors, such as UV exposure and perspiration, to further elucidate their roles in shaping skin microbial dynamics.

Correlation analyses revealed significant associations between microbial diversity and environmental/lifestyle factors, which provide important and valuable insights into how external conditions and personal habits influence the skin microbiome during Antarctic expeditions. Notably, a negative correlation was observed between *C. acnes* and sunblock usage in Host A, suggesting that the sunblock components may alter skin conditions, potentially creating a less favorable environment for certain bacterial species [69]. This supports the hypothesis that sunblock, by affecting skin surface properties such as pH or moisture retention, can modulate microbial communities [22]. Components like zinc oxide or titanium dioxide in sunblock may inhibit microbial growth or alter the skin’s chemical microenvironment [70]. Similarly, in Host B, cosmetic usage negatively correlated with *S. agalactiae* and positively with *C. acnes*. This indicates that certain components in cosmetics or sunblock, such as emollients or preservatives, may affect the skin microbial landscape [71,72]. The regular and frequent use of cosmetics may alter the skin characteristics, such as hydration levels [73] or barrier function [74,75]. These findings reinforce the idea that personal care routines can significantly modulate skin microbial communities, especially in environments where external stressors are magnified.

Despite the rich insights, this study has limitations. The small sample size and short expedition duration limit the generalizability of our findings. Expanding the study to include more participants over longer durations would provide a more comprehensive understanding of microbial dynamics. The inclusion of only male participants also precludes exploration of gender-based differences in microbial dynamics, which are known to exist due to hormonal influences, sebum production, and skin physiology [59,76,77,78,79,80]. Additionally, unmeasured variables such as diet, skin hydration, or specific cosmetic ingredients may have influenced microbial outcomes [4,73,81]. These factors represent critical variables that need to be incorporated into future research to provide a more holistic understanding of microbial dynamics. While batch correction was necessary to minimize technical variability, it may have introduced biases in microbial abundance estimates, particularly for low-abundant microbes [32,82]. These limitations highlight the need for caution in generalizing the findings and underscore the importance of addressing these issues in future research to enhance the reliability and applicability of the results.

Understanding the skin microbiome’s response to extreme environments is important for health management in isolated settings, such as during long-duration space missions or expeditions to remote locations. Strategies such as the application of barrier-enhancing formulations or targeted probiotics could help maintain microbial stability in future expeditions [21,83,84,85,86]. Future studies should include larger, more diverse cohorts (e.g., participants of different genders, ages, and skin types), and longer observation periods are needed to validate and extend these findings. Additionally, application of more comprehensive sampling strategies that can cover various skin regions and utilize advanced sequencing techniques would provide a broader understanding of the skin microbiome dynamics [87]. Utilizing multi-omics techniques, such as metagenomics and metatranscriptomics, could offer deeper insights into the functional roles of microbial communities and their interactions with the host [88]. Longitudinal studies are also needed to investigate the cumulative effects of repeated exposure to extreme environments to assess long-term impacts on skin microbial ecosystem formation over time. By addressing these limitations, future research can better inform interventions to preserve microbial homeostasis and promote health in extreme or isolated environments.

## 5. Conclusions

By tracking individual-specific microbial trajectories during Antarctic field missions, this study reveals how extreme environmental exposure, combined with lifestyle constraints, can drive distinct alterations in the skin microbiome. While limited by a small and homogeneous sample and a short study period, our findings highlight not only the microbiome’s sensitivity to external stressors but also further emphasize the importance of host-specific factors in modulating microbial responses. To advance this field, future investigations should adopt longitudinal designs with larger and more diverse populations to unravel the long-term consequences of extreme environments on skin microbial ecology. Such efforts will be essential in developing microbiome-informed strategies to safeguard skin health during polar expeditions, long-duration space travel, and other physiologically demanding settings.

## Figures and Tables

**Table 1:** Average relative abundance (unit: %) at the phylum level in Host A and Host B across different stages of the Antarctic expedition.

Phylum	Host A					Host B			
	BeforeTrip	OnTheWay	Staying	OnTheWayBack	AfterTrip	BeforeTrip	OnTheWay	Staying	AfterTrip
p__Actinomycetota	91.97	91.70	76.12	80.52	61.68	74.58	83.93	80.51	68.66
p__Pseudomonadota	2.65	2.50	10.37	8.57	11.90	9.85	5.44	8.67	14.41
p__Bacillota_D	3.27	3.48	8.25	7.61	17.54	12.90	6.79	5.98	7.34
p__Bacteroidota	1.06	0.71	2.86	1.83	5.51	0.87	1.58	2.46	4.96
p__Bacillota_A	0.44	0.85	0.89	0.49	2.39	1.29	1.14	0.91	4.39
p__Bacillota_C	0.11	0.29	0.62	0.30	0.44	0.19	0.43	0.39	0.24
p__Cyanobacteria	0.02	0.02	0.29	0.01	0.02	0.00	0.00	0.44	0.00
p__Deinococcota	0.00	0.00	0.18	0.17	0.13	0.04	0.03	0.23	0.00
p__Fusobacteriota	0.01	0.01	0.06	0.05	0.12	0.00	0.20	0.06	0.00
p__Planctomycetota	0.00	0.00	0.00	0.00	0.00	0.00	0.00	0.05	0.00
p__Eremiobacterota	0.00	0.00	0.00	0.00	0.00	0.00	0.00	0.02	0.00
p__Gemmatimonadota	0.00	0.00	0.00	0.00	0.00	0.00	0.00	0.02	0.00
Unclassified	0.47	0.43	0.36	0.45	0.28	0.29	0.45	0.28	0.00

## Data Availability

Illumina MiSeq 16S rRNA gene sequence data generated in this study are publicly available in the NCBI Sequence Read Archive (SRA) under accession numbers SRR7188089-SRR7188143 (BioProject ID: PRJNA471938), accessible at https://www.ncbi.nlm.nih.gov/sra.

## Supplementary Materials

The following supporting information can be downloaded at https://www.mdpi.com/article/10.3390/microorganisms13112491/s1, Figure S1: Temporal shifts in bacterial composition at the class and genus levels during the Antarctic expeditions. Figure S2: Alpha diversity measures (Chao1 and Observed Features) across different trip statuses. Figure S3: Resilience Index (RI) for Host A and Host B across five trip statuses: BeforeTrip (Baseline), OnTheWay, Staying, OnTheWayBack, and AfterTrip. Figure S4: Temporal changes in skin microbial species and their correlations with environmental and lifestyle factors in Host A and Host B. Table S1: Average relative abundance (unit: %) at the class level in Host A and Host B across different stages of the Antarctic expedition. Table S2: Top 20 genus-level relative abundances (unit: %) in Host A and Host B across different stages of the Antarctic expedition. Table S3: Average relative abundance (unit: %) at the species level in Host A and Host B across different stages of the Antarctic expedition.
