# Supplementary material for: Case Study on Shifts in Human Skin Microbiome During Antarctica Expeditions
Source: Microorganisms. 2025 Oct 30;13(11):2491. doi: 10.3390/microorganisms13112491 (PMC12654554; doi:10.3390/microorganisms13112491)
Supplement: Supplementary file 1 [file microorganisms-13-02491-s001.zip › Supplementary_Data.pdf]

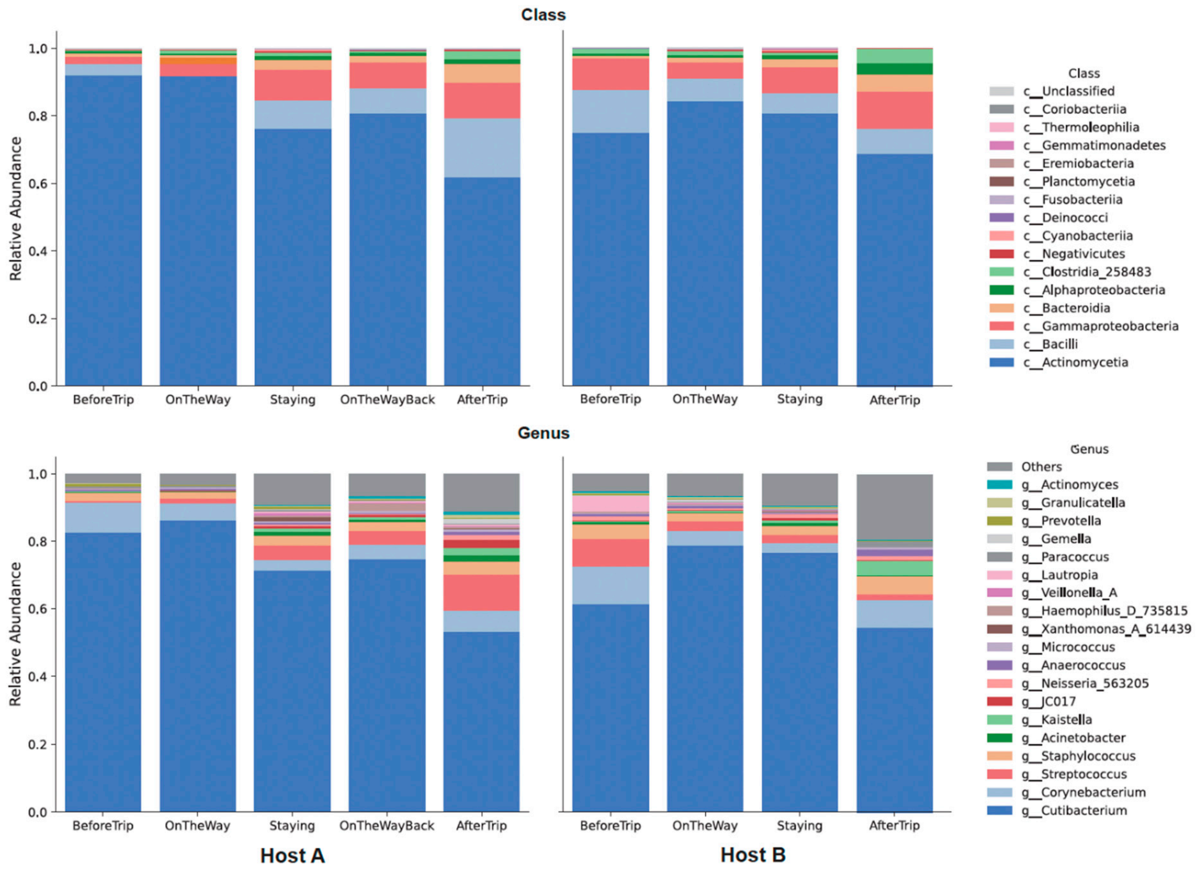

Figure S1. Temporal shifts in bacterial composition at the class and genus levels during the Antarctic expeditions. Relative abundance of bacterial taxa at the class and genus level for Host A and Host B across different stages of the expedition (BeforeTrip, OnTheWay, Staying, OnTheWayBack, AfterTrip).

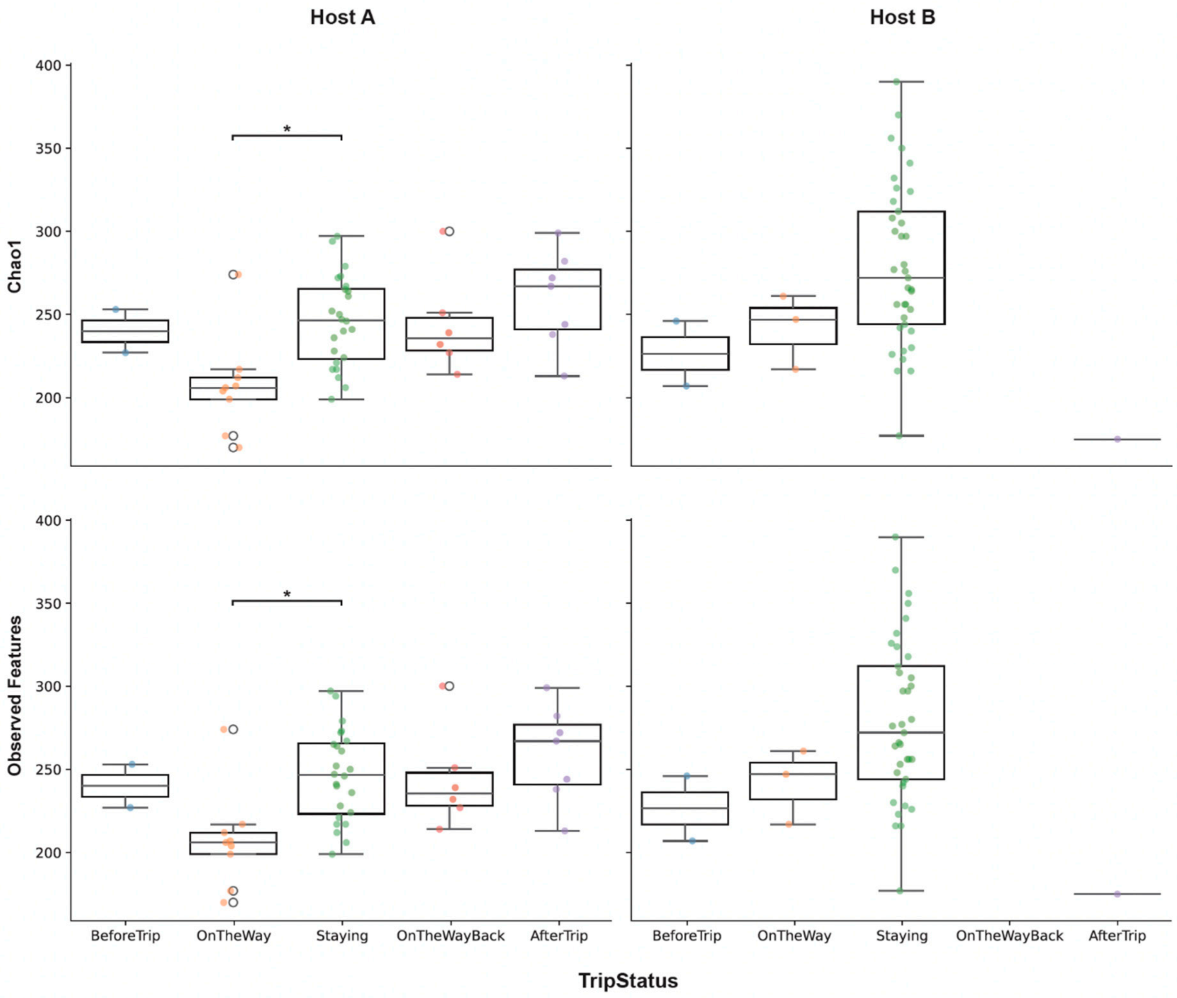

Figure S2. Alpha diversity measures (Chao1 and Observed Features) across different trip statuses. Chao1 and observed features indices for Host A and Host B across different trip stages (BeforeTrip, OnTheWay, Staying, OnTheWayBack, AfterTrip). Significant differences between trip statuses for both hosts were assessed using the Kruskal–Wallis test followed by Bonferroni post hoc correction (ns > 0.05, * $p < 0.05$, ** $p < 0.01$, *** $p < 0.001$, **** $p < 0.0001$).

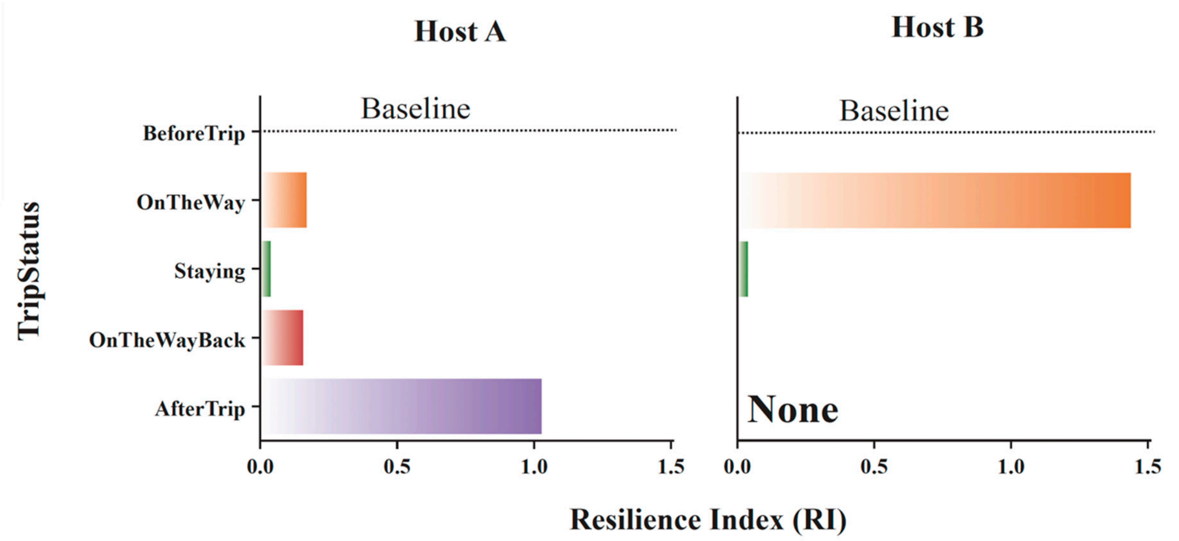

Figure S3. Resilience Index (RI) for Host A and Host B across five trip statuses: BeforeTrip (Baseline), OnTheWay, Staying, OnTheWayBack, and AfterTrip. The dashed line represents the Baseline RI established BeforeTrip. Data was reported as 'None' for Host B during the OnTheWayBack and AfterTrip phases, indicating that RI could not be measured or was zero during these periods.

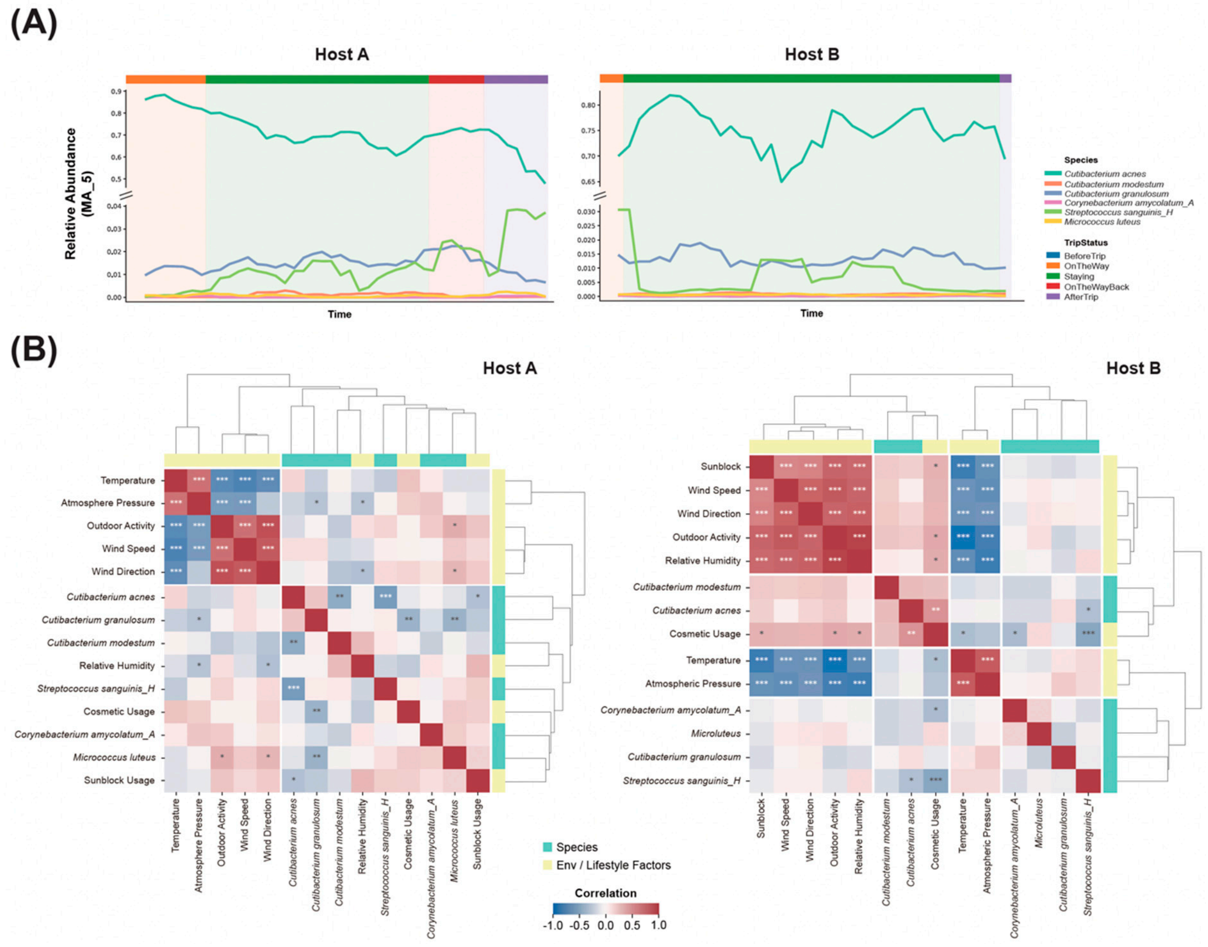

Figure S4. Temporal changes in skin microbial species and their correlations with

environmental and lifestyle factors in Host A and Host B. (A) Moving average calculated across 5 time points of relative abundance for key skin-associated microbial species over time. Different trip statuses are marked by shaded regions, illustrating the temporal dynamics in microbial composition during the expeditions. (B) Correlation matrices between key microbial species and environmental/lifestyle factors for both hosts. Positive correlations (red) and negative correlations (blue) are represented, with significant associations marked (ns > 0.05, * $p < 0.05$, ** $p < 0.01$, *** $p < 0.001$, **** $p < 0.0001$).

Table S1. Average relative abundance (unit: %) at the class level in Host A and Host B across different stages of the Antarctic expedition.

Class	Host A					Host B			
	BeforeTrip	OnTheWay	Staying	OnTheWayBack	AfterTrip	BeforeTrip	OnTheWay	Staying	AfterTrip
**c__Actinomycetia**	91.97	91.70	76.12	80.52	61.68	74.58	83.93	80.49	68.66
**c__Bacilli**	3.27	3.48	8.25	7.61	17.54	12.90	6.79	5.98	7.34
**c__Gammaproteobacteria**	2.00	1.93	9.05	7.63	10.36	9.13	4.70	7.49	11.08
**c__Bacteroidia**	1.06	0.71	2.86	1.83	5.51	0.87	1.58	2.46	4.96
**c__Alphaproteobacteria**	0.65	0.57	1.32	0.93	1.53	0.72	0.75	1.17	3.33
**c__Clostridia_258483**	0.44	0.85	0.89	0.49	2.39	1.29	1.14	0.91	4.39
**c__Negativicutes**	0.11	0.29	0.62	0.30	0.44	0.19	0.43	0.39	0.24
**c__Cyanobacteriia**	0.02	0.02	0.29	0.01	0.02	0.00	0.00	0.44	0.00
**c__Deinococci**	0.00	0.00	0.18	0.17	0.13	0.04	0.03	0.23	0.00
**c__Fusobacteriia**	0.01	0.01	0.06	0.05	0.12	0.00	0.20	0.06	0.00
**c__Planctomycetia**	0.00	0.00	0.00	0.00	0.00	0.00	0.00	0.05	0.00
**c__Eremiobacteria**	0.00	0.00	0.00	0.00	0.00	0.00	0.00	0.02	0.00
**c__Gemmatimonadetes**	0.00	0.00	0.00	0.00	0.00	0.00	0.00	0.02	0.00
**c__Thermoleophilia**	0.00	0.00	0.00	0.00	0.00	0.00	0.00	0.01	0.00
**c__Coriobacteriia**	0.00	0.00	0.00	0.00	0.00	0.00	0.00	0.01	0.00
**Unclassified**	0.47	0.43	0.36	0.45	0.28	0.29	0.45	0.28	0.00

Table S2. Top 20 genus-level relative abundances (unit: %) in Host A and Host B across different stages of the Antarctic expedition.

Genus	Host A					Host B			
	BeforeTrip	OnTheWay	Staying	OnTheWayBack	AfterTrip	BeforeTrip	OnTheWay	Staying	AfterTrip
**g__Cutibacterium**	82.59	86.10	71.18	74.52	53.02	61.17	78.69	76.45	54.29
**g__Corynebacterium**	8.75	5.09	3.22	4.42	6.17	11.35	4.22	2.91	8.17
**g__Streptococcus**	0.59	1.33	4.32	3.93	10.84	8.12	2.93	2.48	1.79
**g__Staphylococcus**	2.40	2.02	2.69	2.79	3.78	4.24	2.51	2.46	5.49
**g__Acinetobacter**	0.18	0.09	1.22	0.74	2.05	0.81	0.13	1.16	0.16
**g__Kaistella**	0.10	0.14	0.99	0.64	2.08	0.13	0.44	0.60	4.30
**g__JC017**	0.03	0.19	0.74	0.66	2.39	0.15	0.33	0.80	0.37
**g__Neisseria_563205**	0.18	0.02	0.44	0.34	1.41	1.29	0.53	1.05	1.08
**g__Anaerococcus**	0.33	0.52	0.45	0.29	0.91	0.65	0.61	0.37	2.02
**g__Micrococcus**	0.36	0.29	0.54	0.56	0.85	0.23	0.24	0.30	0.57
**g__Xanthomonas_A_614439**	0.00	0.00	1.29	0.03	0.21	0.01	0.05	0.12	0.00
**g__Haemophilus_D_735815**	0.10	0.01	0.48	2.17	0.41	0.52	0.17	0.15	0.07
**g__Veillonella_A**	0.10	0.17	0.61	0.26	0.38	0.14	0.32	0.34	0.00
**g__Lautropia**	0.00	0.05	0.54	0.53	0.28	4.38	0.13	0.11	0.00
**g__Paracoccus**	0.49	0.34	0.50	0.11	0.35	0.19	0.25	0.19	1.79
**g__Gemella**	0.03	0.04	0.26	0.15	1.55	0.17	0.73	0.19	0.00
**g__Prevotella**	0.61	0.12	0.54	0.05	0.10	0.35	0.37	0.25	0.05
**g__Granulicatella**	0.22	0.01	0.27	0.38	0.98	0.22	0.38	0.24	0.00
**g__Actinomyces**	0.05	0.05	0.36	0.58	0.90	0.49	0.21	0.14	0.42
**Unclassified**	2.26	2.17	4.87	3.82	4.91	2.24	3.92	4.96	11.12

Table S3. Average relative abundance (unit: %) at the species level in Host A and Host B across different stages of the Antarctic expedition.

Species	Host A					Host B			
	BeforeTrip	OnTheWay	Staying	OnTheWayBack	AfterTrip	BeforeTrip	OnTheWay	Staying	AfterTrip
s__Cutibacterium acnes	81.65	84.94	69.32	72.86	52.18	59.67	77.15	75.05	52.50
s__Cutibacterium granulosum	0.81	1.14	1.70	1.63	0.81	1.49	1.42	1.31	1.70
s__Streptococcus sanguinis_H	0.06	0.17	1.13	1.83	3.32	7.16	0.34	0.53	0.16
s__JC017 sp004296775	0.03	0.19	0.74	0.66	2.39	0.15	0.33	0.80	0.37
s__Kaistella haifensis	0.06	0.10	0.82	0.52	1.89	0.12	0.29	0.41	3.69
s__Lautropia mirabilis	0.00	0.05	0.54	0.53	0.28	4.38	0.13	0.11	0.00
s__Cloacibacterium normanense	0.00	0.14	0.21	0.25	0.36	0.06	0.32	0.29	0.02
s__Finegoldia magna_H	0.04	0.12	0.19	0.09	1.02	0.30	0.29	0.13	1.59
s__Paracoccus xiamenensis	0.30	0.34	0.34	0.10	0.14	0.08	0.07	0.11	1.79
s__Brevundimonas nasdae_A_487984	0.05	0.04	0.22	0.24	0.18	0.22	0.06	0.27	0.01
s__Anaerococcus nagyae	0.31	0.18	0.19	0.08	0.12	0.13	0.10	0.12	0.18
s__Anaerococcus octavius	0.01	0.09	0.15	0.19	0.37	0.20	0.18	0.09	0.31
s__Acidocella facilis	0.03	0.07	0.11	0.16	0.28	0.15	0.10	0.11	0.00
s__Corynebacterium durum	0.00	0.01	0.13	0.21	0.35	0.68	0.07	0.05	0.29
s__Peptoniphilus_A lacydonensis	0.04	0.07	0.16	0.04	0.31	0.17	0.11	0.06	0.32
s__Corynebacterium accolens	0.04	0.06	0.08	0.06	0.46	0.02	0.02	0.07	0.05
s__Prevotella melaninogenica	0.40	0.00	0.20	0.00	0.07	0.01	0.08	0.07	0.00

**s__SIO2C1 sp010672925**	**0.00**	**0.00**	**0.12**	**0.00**	**0.00**	**0.00**	**0.00**	**0.14**	**0.00**
**s__Cutibacterium modestum**	**0.13**	**0.02**	**0.16**	**0.03**	**0.03**	**0.01**	**0.10**	**0.08**	**0.09**
**Unclassified**	**15.36**	**11.65**	**22.28**	**19.48**	**33.92**	**21.82**	**17.45**	**18.64**	**35.88**
