# Supplementary material for: Case Study on Shifts in Human Skin Microbiome During Antarctica Expeditions
Source: Microorganisms. 2025 Oct 30;13(11):2491. doi: 10.3390/microorganisms13112491 (PMC12654554; doi:10.3390/microorganisms13112491)
Supplement: Supplementary file 1 [file microorganisms-13-02491-s001.zip › Table_S1.pdf]

Table S1. Average relative abundance (unit: %) at the class level in Host A and Host B across different stages of the Antarctic expedition.

Class	Host A					Host B			
	BeforeTrip	OnTheWay	Staying	OnTheWayBack	AfterTrip	BeforeTrip	OnTheWay	Staying	AfterTrip
c__Actinomycetia	91.97	91.70	76.12	80.52	61.68	74.58	83.93	80.49	68.66
c__Bacilli	3.27	3.48	8.25	7.61	17.54	12.90	6.79	5.98	7.34
c__Gammaproteobacteria	2.00	1.93	9.05	7.63	10.36	9.13	4.70	7.49	11.08
c__Bacteroidia	1.06	0.71	2.86	1.83	5.51	0.87	1.58	2.46	4.96
c__Alphaproteobacteria	0.65	0.57	1.32	0.93	1.53	0.72	0.75	1.17	3.33
c__Clostridia_258483	0.44	0.85	0.89	0.49	2.39	1.29	1.14	0.91	4.39
c__Negativicutes	0.11	0.29	0.62	0.30	0.44	0.19	0.43	0.39	0.24
c__Cyanobacteriia	0.02	0.02	0.29	0.01	0.02	0.00	0.00	0.44	0.00
c__Deinococci	0.00	0.00	0.18	0.17	0.13	0.04	0.03	0.23	0.00
c__Fusobacteriia	0.01	0.01	0.06	0.05	0.12	0.00	0.20	0.06	0.00
c__Planctomycetia	0.00	0.00	0.00	0.00	0.00	0.00	0.00	0.05	0.00
c__Eremiobacteria	0.00	0.00	0.00	0.00	0.00	0.00	0.00	0.02	0.00
c__Gemmatimonadetes	0.00	0.00	0.00	0.00	0.00	0.00	0.00	0.02	0.00
c__Thermoleophilia	0.00	0.00	0.00	0.00	0.00	0.00	0.00	0.01	0.00
c__Coriobacteriia	0.00	0.00	0.00	0.00	0.00	0.00	0.00	0.01	0.00
Unclassified	0.47	0.43	0.36	0.45	0.28	0.29	0.45	0.28	0.00
