# Supplementary material for: Case Study on Shifts in Human Skin Microbiome During Antarctica Expeditions
Source: Microorganisms. 2025 Oct 30;13(11):2491. doi: 10.3390/microorganisms13112491 (PMC12654554; doi:10.3390/microorganisms13112491)
Supplement: Supplementary file 1 [file microorganisms-13-02491-s001.zip › Table_S2.pdf]

Table S2. Top 20 genus-level relative abundances (unit: %) in Host A and Host B across different stages of the Antarctic expedition.

Genus	Host A					Host B			
	BeforeTrip	OnTheWay	Staying	OnTheWayBack	AfterTrip	BeforeTrip	OnTheWay	Staying	AfterTrip
**g__Cutibacterium**	82.59	86.10	71.18	74.52	53.02	61.17	78.69	76.45	54.29
**g__Corynebacterium**	8.75	5.09	3.22	4.42	6.17	11.35	4.22	2.91	8.17
**g__Streptococcus**	0.59	1.33	4.32	3.93	10.84	8.12	2.93	2.48	1.79
**g__Staphylococcus**	2.40	2.02	2.69	2.79	3.78	4.24	2.51	2.46	5.49
**g__Acinetobacter**	0.18	0.09	1.22	0.74	2.05	0.81	0.13	1.16	0.16
**g__Kaistella**	0.10	0.14	0.99	0.64	2.08	0.13	0.44	0.60	4.30
**g__JC017**	0.03	0.19	0.74	0.66	2.39	0.15	0.33	0.80	0.37
**g__Neisseria_563205**	0.18	0.02	0.44	0.34	1.41	1.29	0.53	1.05	1.08
**g__Anaerococcus**	0.33	0.52	0.45	0.29	0.91	0.65	0.61	0.37	2.02
**g__Micrococcus**	0.36	0.29	0.54	0.56	0.85	0.23	0.24	0.30	0.57
**g__Xanthomonas_A_614439**	0.00	0.00	1.29	0.03	0.21	0.01	0.05	0.12	0.00
**g__Haemophilus_D_735815**	0.10	0.01	0.48	2.17	0.41	0.52	0.17	0.15	0.07
**g__Veillonella_A**	0.10	0.17	0.61	0.26	0.38	0.14	0.32	0.34	0.00
**g__Lautropia**	0.00	0.05	0.54	0.53	0.28	4.38	0.13	0.11	0.00
**g__Paracoccus**	0.49	0.34	0.50	0.11	0.35	0.19	0.25	0.19	1.79
**g__Gemella**	0.03	0.04	0.26	0.15	1.55	0.17	0.73	0.19	0.00
**g__Prevotella**	0.61	0.12	0.54	0.05	0.10	0.35	0.37	0.25	0.05
**g__Granulicatella**	0.22	0.01	0.27	0.38	0.98	0.22	0.38	0.24	0.00
**g__Actinomyces**	0.05	0.05	0.36	0.58	0.90	0.49	0.21	0.14	0.42
**Unclassified**	2.26	2.17	4.87	3.82	4.91	2.24	3.92	4.96	11.12
