# Supplementary material for: Case Study on Shifts in Human Skin Microbiome During Antarctica Expeditions
Source: Microorganisms. 2025 Oct 30;13(11):2491. doi: 10.3390/microorganisms13112491 (PMC12654554; doi:10.3390/microorganisms13112491)
Supplement: Supplementary file 1 [file microorganisms-13-02491-s001.zip › Table_S3.pdf]

Table S3. Average relative abundance (unit: %) at the species level in Host A and Host B across different stages of the Antarctic expedition.

Species	Host A					Host B			
	BeforeTrip	OnTheWay	Staying	OnTheWayBack	AfterTrip	BeforeTrip	OnTheWay	Staying	AfterTrip
s__Cutibacterium acnes	81.65	84.94	69.32	72.86	52.18	59.67	77.15	75.05	52.50
s__Cutibacterium granulosum	0.81	1.14	1.70	1.63	0.81	1.49	1.42	1.31	1.70
s__Streptococcus sanguinis_H	0.06	0.17	1.13	1.83	3.32	7.16	0.34	0.53	0.16
s__JC017 sp004296775	0.03	0.19	0.74	0.66	2.39	0.15	0.33	0.80	0.37
s__Kaistella haifensis	0.06	0.10	0.82	0.52	1.89	0.12	0.29	0.41	3.69
s__Lautropia mirabilis	0.00	0.05	0.54	0.53	0.28	4.38	0.13	0.11	0.00
s__Cloacibacterium normanense	0.00	0.14	0.21	0.25	0.36	0.06	0.32	0.29	0.02
s__Finegoldia magna_H	0.04	0.12	0.19	0.09	1.02	0.30	0.29	0.13	1.59
s__Paracoccus xiamenensis	0.30	0.34	0.34	0.10	0.14	0.08	0.07	0.11	1.79
s__Brevundimonas nasdae_A_487984	0.05	0.04	0.22	0.24	0.18	0.22	0.06	0.27	0.01
s__Anaerococcus nagyae	0.31	0.18	0.19	0.08	0.12	0.13	0.10	0.12	0.18
s__Anaerococcus octavius	0.01	0.09	0.15	0.19	0.37	0.20	0.18	0.09	0.31
s__Acidocella facilis	0.03	0.07	0.11	0.16	0.28	0.15	0.10	0.11	0.00
s__Corynebacterium durum	0.00	0.01	0.13	0.21	0.35	0.68	0.07	0.05	0.29
s__Peptoniphilus_A lacydonensis	0.04	0.07	0.16	0.04	0.31	0.17	0.11	0.06	0.32
s__Corynebacterium accolens	0.04	0.06	0.08	0.06	0.46	0.02	0.02	0.07	0.05
s__Prevotella melaninogenica	0.40	0.00	0.20	0.00	0.07	0.01	0.08	0.07	0.00

**s__SIO2C1 sp010672925**	**0.00**	**0.00**	**0.12**	**0.00**	**0.00**	**0.00**	**0.00**	**0.14**	**0.00**
**s__Cutibacterium modestum**	**0.13**	**0.02**	**0.16**	**0.03**	**0.03**	**0.01**	**0.10**	**0.08**	**0.09**
**Unclassified**	**15.36**	**11.65**	**22.28**	**19.48**	**33.92**	**21.82**	**17.45**	**18.64**	**35.88**
